# Revisiting pediatric HGGs and PNETs according to the WHO CNS5 criteria: A clinical and genomic retrospective analysis

**DOI:** 10.1093/noajnl/vdaf175

**Published:** 2025-08-09

**Authors:** Joo Whan Kim, Seung Ah Choi, Sungyoung Lee, Hongseok Yun, Ji Hoon Phi, Sung-Hye Park, Seung-Ki Kim

**Affiliations:** Department of Genomic Medicine, Seoul National University Hospital, Seoul, Republic of Korea; Division of Pediatric Neurosurgery, Seoul National University Children’s Hospital, Seoul National University College of Medicine, Seoul, Republic of Korea; Division of Pediatric Neurosurgery, Seoul National University Children’s Hospital, Seoul National University College of Medicine, Seoul, Republic of Korea; Healthcare AI Research Institute, Seoul National University Hospital, Seoul, Republic of Korea; Department of Genomic Medicine, Seoul National University Hospital, Seoul, Republic of Korea; Department of Genomic Medicine, Seoul National University Hospital, Seoul, Republic of Korea; Neuroscience Institute, Seoul National University College of Medicine, Seoul, Republic of Korea; Division of Pediatric Neurosurgery, Seoul National University Children’s Hospital, Seoul National University College of Medicine, Seoul, Republic of Korea; Department of Pathology, Seoul National University Children’s Hospital, Seoul National University College of Medicine, Seoul, Republic of Korea; Neuroscience Institute, Seoul National University College of Medicine, Seoul, Republic of Korea; Neuroscience Institute, Seoul National University College of Medicine, Seoul, Republic of Korea; Division of Pediatric Neurosurgery, Seoul National University Children’s Hospital, Seoul National University College of Medicine, Seoul, Republic of Korea

**Keywords:** cancer predisposition syndrome, diffuse high-grade glioma, next-generation sequencing, outcome, pediatric

## Abstract

**Background:**

The 2021 WHO Classification of Tumors of the Central Nervous System, 5th edition (WHO CNS5), introduced revised diagnostic criteria for pediatric brain tumors (BTs), redefining pediatric-type diffuse high-grade gliomas (pHGGs) into 4 subtypes: diffuse midline glioma, H3 K27-altered (DMG-H3K27), diffuse hemispheric glioma, H3 G34-mutant (DHG-H3G34), diffuse pediatric-type high-grade glioma, H3-wildtype and IDH-wildtype (DpHGG-H3wt/IDHwt), and infant-type hemispheric glioma (IHG). This study revisits prior diagnoses of HGGs and primitive neuroectodermal tumors (PNETs) in children and incorporates next-generation sequencing (NGS) to classify tumors according to the revised criteria and analyze their clinicogenomic characteristics and outcomes.

**Methods:**

A retrospective review of pediatric patients diagnosed with glioblastoma (GBM), anaplastic astrocytoma, anaplastic oligoastrocytoma (AOA), gliomatosis cerebri, or PNET between 1997 and 2023 was conducted. Cases underwent pathology review, immunohistochemistry (IHC), and BT-targeted NGS for reclassification per WHO CNS5. An additional 20 patients diagnosed with pHGG via genetics-integrated diagnosis since 2020 were included. Clinical characteristics, genomic alterations, and outcomes were analyzed.

**Results:**

Among the 78 reviewed cases, 41 were reclassified as pHGGs. *TP53* mutations were the most prevalent, particularly in DpHGG-H3wt/IDHwt, which showed associations with cancer predisposition syndrome (CPS). Two patients with Li-Fraumeni syndrome (LFS) developed DpHGG-H3wt/IDHwt adjacent to prior radiation fields. The 2-year overall survival (OS) rates were lowest in DpHGG-H3wt/IDHwt (23.2%) and highest in IHG (92.3%). Long-term survival was observed in IHG patients, with a 5-year OS rate of 73.8%, indicating the need for different adjuvant treatment strategies compared to other pHGGs.

**Conclusions:**

BT-targeted NGS facilitates the reclassification of pHGGs, revealing associations with CPS. Routine germline sequencing is warranted, and accurate molecular diagnosis enables a shift in treatment strategies tailored to specific pHGG subtypes.

**Key Points**

- Diffuse pediatric-type high-grade gliomas (HGGs), H3-wildtype, and IDH-wildtype (DpHGG-H3wt/IDHwt) showed a significant association with cancer predisposition syndrome (CPS).
- Routine germline sequencing is recommended for pediatric-type HGG (pHGG) patients to identify underlying CPS.
- Reclassification of pHGG revealed the favorable prognosis of IHG, necessitating a revision of previous therapeutic approaches.

**Importance of the Study**

This study provides insights into the molecular and clinical characteristics of pediatric high-grade gliomas (pHGGs) via next-generation sequencing (NGS) and the revised WHO CNS5 classification. The findings emphasize the necessity of reevaluating historical diagnoses to improve our understanding of the diseases. Notably, the association between DpHGG-H3wt/IDHwt and CPS underscores the need for routine germline sequencing in affected children. These results can guide future therapeutic strategies and genetic counseling efforts, ultimately contributing to enhanced prognosis and individualized treatment approaches for pHGG patients.

With the emergence of genetic sequencing, various types of molecular hallmarks have been integrated into the diagnosis of brain tumors (BTs) in the 2021 5th edition of the WHO classification of tumors of the central nervous system (CNS) (WHO CNS5).^1,2^ Notably, for pediatric-type BTs, former diagnostic terms such as anaplastic astrocytoma (AA), glioblastoma (GBM) or primitive neuroectodermal tumor (PNET) are no longer used.^1,3,4^ The term “pediatric-type” glioma first appeared in the WHO CNS5, and pediatric-type diffuse high-grade glioma (pHGG) is now classified into 4 distinct disease entities: diffuse midline glioma, H3 K27-altered (DMG-H3K27), diffuse hemispheric glioma, H3 G34-mutant (DHG-H3G34), diffuse pediatric-type high-grade glioma, H3-wildtype and IDH-wildtype (DpHGG-H3wt/IDHwt), and infant-type hemispheric glioma (IHG).^5–8^

Although the revised diagnostic criteria have clarified the disease characteristics, the pHGG category comprises subtypes with various biological features. With the increasing use of next-generation sequencing (NGS) and methylation assays, further revision of this diagnostic entity is likely inevitable in future WHO classifications of CNS tumors.^9^

As the volume of sequencing data continues to grow, multicenter, open-source tumor genetic databases are being established.^10,11^ Efforts are also being made to create a comprehensive pediatric cancer database as a national task.^12,13^ However, due to the rarity of pediatric CNS tumors, a large-cohort genetic database for these tumors has not been fully developed. Additionally, the updates and evolving classifications of pediatric CNS tumors, as described above, complicate the understanding of the disease when considered with clinical information from previous patient cohorts.^14^

We reported the clinical outcomes of children with HGGs at our center, which were distinct from those of adult HGGs.^15,16^ However, the clinical outcomes of newly classified pHGGs according to the WHO CNS5 criteria remain uncertain. This study reevaluates the previous diagnoses of HGGs and PNETs in children through pathology review, immunohistochemistry (IHC), and BT-targeted NGS of tumor tissues. Additionally, we included patients diagnosed with DMG-H3K27, DHG-H3G34, DpHGG-H3wt/IDHwt, and IHG through genetics-integrated diagnosis according to the WHO CNS5 after the introduction of BT-targeted NGS to integrate our pHGG data. Our aim was to understand the clinicogenomic characteristics and prognoses of these tumors according to the revised WHO CNS5.

## Methods

### Patients

This retrospective study included patients who underwent surgery at Seoul National University Children’s Hospital. The study period spans from 1997 to 2023, including an additional 20 pHGG patients diagnosed after January 2020. These patients had pathologically confirmed diagnoses of GBM, AA, anaplastic oligoastrocytoma (AOA), gliomatosis cerebri (GC), or PNET. Among the 87 patients initially diagnosed during this period, 9 were excluded because of a lack of archived tumor tissues required for further IHC or BT-targeted NGS.

Seventy-eight eligible patients were reclassified according to the WHO CNS5 criteria. In addition to pHGG, cases were reclassified into other tumor types, including supratentorial ependymoma (ST-EPN), atypical teratoid/rhabdoid tumor (AT/RT), and low-grade gliomas. To enhance the analysis of the clinical characteristics of pHGGs, 20 additional patients diagnosed with pHGGs between January 2020 and November 2023 were included. BT-targeted NGS has been performed at our institution since 2018; therefore, these additional cases could be diagnosed according to the WHO CNS5. Clinicogenomic analysis was performed for 61 pHGG patients (Supplementary Figure 1).

The clinical characteristics, pathological findings, and IHC results of the patients were reviewed. Patients underwent safe maximal surgical resection whenever feasible or biopsy if the tumor was in the brainstem or was too infiltrative to be resected. The extent of resection was classified as follows: gross total resection (GTR), no residual tumor on postoperative MRI; subtotal resection (STR), >90% of tumor volume resected on MRI; partial resection, >50% of tumor volume resected; and biopsy, <50% of the tumor volume resected. Adjuvant chemotherapy and radiotherapy (RT) were administered on the basis of the previous diagnoses of HGG and PNET. Patients diagnosed with HGG were treated according to the Korean Society for Pediatric Neuro-Oncology-A112 protocol, which involves concomitant chemoradiotherapy (CCRT) with temozolomide as proposed by Stupp et al.,^17^ unless the patient’s condition was too poor or the caregiver refused treatment. RT to the tumor involved field with planning target volume was performed in patients over the age of 3.

This study protocol was approved by the local Institutional Review Board (IRB No. 2303-098-1412) and was conducted in accordance with the Declaration of Helsinki. Informed consent was waived by the IRB because of the retrospective design of the study.

### Histopathology, IHC, and Molecular Diagnosis

Pathology, IHC, and BT-targeted NGS results were reviewed, and the diagnosis was revisited according to the diagnostic criteria of the WHO CNS5. For the IHC study, the primary antibodies were anti-ATRX (1:300, Atlas Antibodies, AB, Bromma, Sweden), anti-GFAP (1:200, 6F2, DAKO, Glostrup, Denmark), anti-H3K27M (1:1,000, Millipore, Temecula, CA), anti-H3K27me3 (1:100, C36B11, Cell Signaling, Boston, MA), antisynaptophysin (1:200, 27G12, Novocastra, Newcastle, UK), anti-IDH1 R132H (1:100, H09, Dianova, Hamburg, Germany), anti-INI-1 (1:100, MRQ27, Cell Marque, Rocklin, CA), anti-Ki-67 (1:1,000, M7240, DAKO), anti-L1CAM (1:10,000, UJ127, Millipore), anti-NFkB (1:1,000, D14E12, Cell Signaling), anti-p53 (1:100, DO7, DAKO), and anti-pHH3 (1:100, Cell Marque). IHC staining was performed via a standard avidin-biotin-peroxidase method using a BenchMark ULTRA system (Roche Diagnostics, Indianapolis, IN). The positive control consisted of known positive tissue and tissue containing entrapped positive cells, whereas the negative control involved omission of the primary antibody. The number of Ki-67 labeled cells was calculated in whole-slide images via the Sectra Ki-67-labeling cell-counting algorithm. The primary antibodies used in this study are listed in Supplementary Table 1.

### BT-Targeted NGS and Data Analysis

Tumor DNA was extracted from the fresh frozen tissue of the patients, and prepared using SureSelect XT (Agilent Technologies, Santa Clara, CA) and SureSelect XT-HS, in accordance with the respective panel versions. In addition, tumor RNA was extracted and prepared using SureSelect XT-HS2 (Agilent Technologies) from the BT panel version 3.2. Paired-end NGS data were subsequently generated via the HiSeq 2500 or NextSeq 550Dx platform (Illumina Inc., San Diego, CA).

The raw sequences of the FASTA files were subjected to adaptor trimming (Trimmomatic version 0.36), followed by alignment to the hg19 reference genome via Burrows-Wheeler Aligner version 0.7.17. The alignment result was further processed for deduplication and base recalibration via GATK version 4.0.2.1. Variant calling was performed by integrating the results from multiple SNV/INDEL calling tools, including MuTect version 1.1.7, IndelGenotyper from GATK version 4.0.2.1, SNVer version 0.5.3, LoFreq version 2.1.2, and VarDict version 1.8.2. For CNV calling, an in-house CNV caller and CNVkit version 0.9.9 were used. Translocation was identified using STAR-Fusion version 1.10.1 and Arriba version 2.1.0.

The identified variants were reviewed by both bioinformatics experts and pathologists through manual curation, literature review, and, when necessary, additional experimental validation. Variants with a frequency greater than 1% in population databases (gnomAD EAS, KRGDB, KOVA, and NARD2) were excluded from further analysis prior to review. The oncogenic effects of the detected variants were evaluated using the OncoKB^TM^ database.^18^

Although BT-targeted NGS was performed on fresh frozen tumor tissues stored for a significant period (14.1 ± 6.5 years), the quality of the sequencing was adequate, as evidenced by a low duplication rate (22.6 ± 4.2%), high mean coverage depth (838.5 ± 250.1×), and high on-target rate (85.2 ± 4.9%) (Supplementary Figure 2).

### Germline Sequencing and Determination of Cancer Predisposition Syndrome

From the BT-targeted NGS results, genes with known germline mutations with a tumor-observed variant allele frequency (VAF) greater than 30% for single-nucleotide variants and greater than 20% for small insertions and deletions were considered for additional germline sequencing. VAF refers to the proportion of a specific variant among the total number of sequencing reads. The genes considered for germline analysis included *TP53*, *NF1*, *MLH1*, *MSH6*, *PMS2*, *PTEN*, *RB1*, and *ATM*.^19^ When matched blood-derived DNA was available, whole-exome sequencing at 50× depth was performed. Libraries were prepared via the SureSelect V4-post kit (Agilent Technologies). Paired-end sequencing was conducted on the Illumina platform.

Almost none of the patients diagnosed before 2020 had matched blood-derived DNA available for germline analysis. This limitation was due to the fact that prior to 2020, the focus of storage was on fresh tumor tissue, and the survival status of pHGG patients made it impossible to obtain additional blood samples. For patients diagnosed after 2020, germline testing was performed when the VAF observed via tumor sequencing met the thresholds described above.

In the absence of matched blood-derived DNA from the patient, the probability of cancer predisposition syndrome (CPS) was determined on the basis of the patient’s cancer history and family history of rare, specific cancers with a young age of onset.^20^ The clinical criteria for Li-Fraumeni syndrome (LFS) were evaluated according to the Chompret criteria.^21^ Neurofibromatosis type 1 (NF-1) was diagnosed on the basis of the revised diagnostic criteria of Legius et al.^22^ The desirable diagnostic criteria for constitutional mismatch repair deficiency (cMMRD) syndrome could be the presence of hypermutated genomic profiles with mutations in one of the 4 mismatch repair genes (*MSH2, MSH6, MLH1*, and *PMS2*) in BTs.^23^ CPS was determined in patients with a germline variant validated through sequencing, combined with the clinical diagnostic criteria.

### Statistical Analysis

R software version 4.4.1 was used for the statistical analysis. To evaluate the statistical significance of differences between categorical variables, the chi-square test for independence or Fisher’s exact test was applied. For continuous variables, the Kruskal–Wallis test was used to analyze age, which is a nonnormally distributed variable. A Kaplan–Meier curve was used to estimate overall survival (OS). A *P*-value less than .05 was considered statistically significant, and all *P*-values were 2-sided. The alluvial plot was generated using the RAWGraphs platform.^24^

## Results

### Revisiting the Previous Diagnosis of pHGGs and PNETs According to the WHO CNS5 Criteria

**Figure 1.**
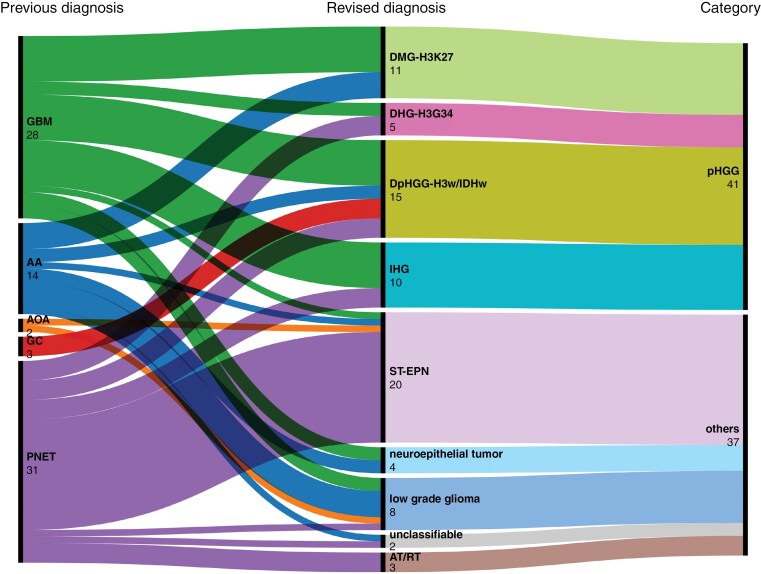
Alluvial plot (https://rawgraphs.io/) for revisiting the previous diagnosis of pHGGs and PNETs according to the WHO CNS5. The cohort includes a total of 78 patients, comprising 41 with pHGG and 37 with other diagnoses. Abbreviations: GBM, glioblastoma; AA, anaplastic astrocytoma; AOA, anaplastic oligoastrocytoma; GC, gliomatosis cerebri; PNET, primitive neuroectodermal tumor; pHGG, pediatric-type diffuse high-grade glioma; DMG-H3K27, diffuse midline glioma, H3 K27-altered; DHG-H3G34, diffuse hemispheric glioma, H3 G34-mutant; DpHGG-H3wt/IDHwt, diffuse pediatric-type high-grade glioma, H3-wildtype and IDH-wildtype; IHG, infant-type hemispheric glioma; ST-EPN, supratentorial ependymoma; AT/RT, atypical teratoid/rhabdoid tumor; WHO CNS5, The 2021 WHO Classification of Tumors of the Central Nervous System, 5th edition.

The diagnoses of 78 patients were revisited by diagnostic review according to the WHO CNS5 criteria. The initial diagnoses were as follows: 28 GBM, 14 AA, 2 AOA, 3 GC, and 31 PNET. Among these, the results of the IHC assessment of H3K27M nuclear expression and loss of H3K27me3 in 3 patients were consistent with DMG-H3K27. BT-targeted NGS was performed using fresh frozen tumor tissues from the remaining 75 patients. The following previously diagnosed cases were reclassified as pHGGs: 82.1% (23/28) of GBMs, 42.9% (6/14) of AAs, 3 (100%) of GCs, and 29.0% (9/31) of PNETs (Figure 1). Among those 41 patients who were reclassified and diagnosed with pHGGs, the distribution was as follows: 11 with DMG-H3K27, 5 with DHG-H3G34, 15 with DpHGG-H3wt/IDHwt, and 10 with IHG. All 3 GC patients were reclassified as DpHGG-H3wt/IDHwt. Among the remaining 37 patients with reclassified diagnoses, ST-EPN was the most common and accounted for 54.0% (20 patients). The remaining diagnoses included 3 AT/RTs, 8 diffuse low-grade gliomas, 4 emerging neuroepithelial tumors, and 2 unclassifiable tumors. The unclassifiable tumors that were initially diagnosed as AA or PNET remained unclassifiable after BT-targeted NGS due to the absence of driver mutations. Their sequencing quality parameters were duplication rates of 31.7% and 30.9%, mean coverage depths of 974.9 × and 1040.7×, and on-target rates of 88.2% and 85.9% respectively.

**Figure 2.**
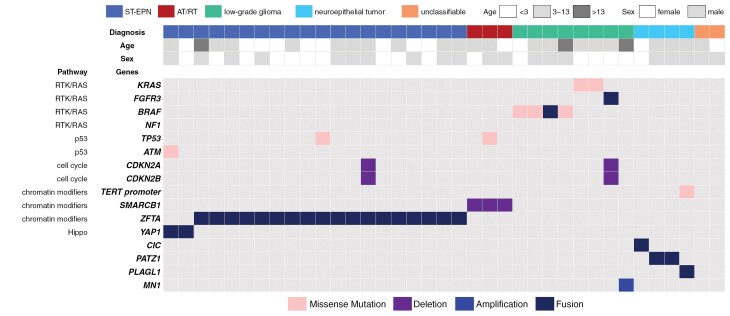
Oncoplot of 37 patients with diagnoses other than pHGG. All patients harbored pathognomonic genetic alterations except for 2 unclassifiable patients. These patients were initially diagnosed with AA or PNET and remained unclassifiable due to the absence of driver mutations according to BT-targeted NGS. Fusion gene detection via transcriptome sequencing facilitated the diagnosis of ST-EPN, low-grade gliomas, and neuroepithelial tumors, as fusion genes were the only observed driver events in these tumors. Abbreviations: ST-EPN, supratentorial ependymoma; AT/RT, atypical teratoid/rhabdoid tumor; pHGG, pediatric-type diffuse high-grade glioma; BT, brain tumor; NGS, next-generation sequencing.

Seventeen of 20 ST-EPN patients (85%) had previously been diagnosed with PNET. Histopathologically, these tumors present as compact sheets of monotonous primitive tumor cells with fine capillaries. However, they lack typical ependymal patterns, such as perivascular pseudorosettes, ependymal rosettes or ependymal canals. ST-EPN patients harbored pathognomonic *ZFTA* or *YAP1* gene fusions, whereas AT/RT patients presented with *SMARCB1* deletions (Figure 2).

The 8 low-grade gliomas harbored alterations in *BRAF, KRAS, FGFR3*, and *MN1*. The diagnoses included 3 pilocytic astrocytomas, 1 diffuse low-grade glioma, MAPK pathway-altered, 1 polymorphous low-grade neuroepithelial tumor of the young, 1 astroblastoma, MN1-altered, 1 desmoplastic infantile astrocytoma, and 1 diffuse leptomeningeal glioneuronal tumor. The 4 emerging neuroepithelial tumors were diagnosed based on detection of the *CIC*, *PATZ1*, and *PLAGL1* fusion genes.

### Clinical Characteristics of pHGGs

Sixty-one pHGG patients were investigated, including 20 patients diagnosed with pHGGs from 2020 to 2023 according to the WHO CNS5 classification (Table 1). The male-to-female ratios were as follows: 1:1.9 in DMG-H3K27 patients, 1:1.3 in DHG-H3G34 patients, 1:0.7 in DpHGG-H3wt/IDHwt patients, and 1:1.7 in IHG patients. The mean age at diagnosis was 10.6 ± 4.7 years in DMG-H3K27 patients, 12.9 ± 2.2 years in DHG-H3G34 patients, 11.2 ± 4.4 years in DpHGG-H3wt/IDHwt patients, and 1.8 ± 1.6 years in IHG patients (Supplementary Figure 3).

**Table 1.** Clinical Characteristics of 61 pHGG Patients

Clinical features	DMG-H3K27 (*n* = 20)	DHG-H3G34 (*n* = 8)	DpHGG-H3wt/IDHwt (*n* = 20)	IHG (*n* = 13)	*P*-value
	Number of cases (%) or value				
Sex					.426
M	7 (35.0%)	3 (37.5%)	12 (60.0%)	6 (46.2%)	
F	13 (65.0%)	5 (62.5%)	8 (40.0%)	7 (53.8%)	
Age (years)					<.001
	10.6 ± 4.7	12.9 ± 2.2	11.2 ± 4.4	1.8 ± 1.6	
Location					<.001
Brainstem	9 (45.0%)	0 (0.0%)	0 (0.0%)	0 (0.0%)	
Thalamus-Basal ganglia	11 (55.0%)	1 (12.5%)	3 (15.0%)	0 (0.0%)	
Lobar	0 (0.0%)	7 (87.5%)	14 (70.0%)	13 (100.0%)	
Diffuse hemispheric	0 (0.0%)	0 (0.0%)	2 (10.0%)	0 (0.0%)	
Spine	0 (0.0%)	0 (0.0%)	1 (5.0%)	0 (0.0%)	
Leptomeningeal seeding at diagnosis					.708
	3 (15.0%)	0 (0.0%)	3 (15.0%)	2 (15.4%)	
Extent of resection					.001
Biopsy & PR	13 (65.0%)	2 (25.0%)	5 (25.0%)	0 (0.0%)	
STR & GTR	7 (35.0%)	6 (75.0%)	15 (75.0%)	13 (100.0%)	
Adjuvant therapy					.001
CCRT	15 (75.0%)	8 (100.0%)	19 (95.0%)	6 (46.2%)	
Radiotherapy only	4 (20.0%)	0 (0.0%)	0 (0.0%)	1 (7.7%)	
Chemotherapy only	0 (0.0%)	0 (0.0%)	0 (0.0%)	5 (38.5%)	
None	1 (5.0%)	0 (0.0%)	1 (5.0%)	1 (7.7%)	
Association with CPS					<.001
	0 (0.0%)	0 (0.0%)	10 (50.0%)	0 (0.0%)	
			5 LFS, 2 NF-1, 3 cMMRD		

Abbreviations: DMG-H3K27, diffuse midline glioma, H3 K27-altered; DHG-H3G34, diffuse hemispheric glioma, H3 G34-mutant; DpHGG-H3wt/IDHwt, diffuse pediatric-type high-grade glioma, H3-wildtype and IDH-wildtype; IHG, infant-type hemispheric glioma; PR, partial resection; STR, subtotal resection; GTR, gross total resection; CRRT, concomitant chemoradiotherapy; CPS, cancer predisposition syndrome; LFS, Li-Fraumeni syndrome; NF-1, neurofibromatosis type 1; cMMRD, constitutional mismatch repair deficiency syndrome.

The tumor location varied significantly among the pHGG subtypes. The epicenters of the DMG-H3K27 tumors included the thalamus and basal ganglia in 11 patients (55.0%) and the brainstem in 9 patients (45.0%). DHG-H3G34 occurred in the frontal lobe (3 patients), temporal lobe (3 patients), and parietal lobe (1 patient), and 1 tumor extended to the midline structure, including the basal ganglia. DpHGG-H3wt/IDHwt occurred in the frontal lobe (9 patients), temporal lobe (4 patients), parietal lobe (1 patient), diffuse hemispheric involvement (2 patients), and cervical spine (1 patient), and 3 tumors involved the midline structure. IHGs occurred in the frontal lobe (7 patients), temporal lobe (2 patients), parietal lobe (2 patients), and occipital lobe (2 patients). No difference was observed in leptomeningeal seeding at diagnosis across tumor types.

The extent of resection varied significantly among the diagnoses, with lower rates of GTR and STR in DMG-H3K27 patients due to the tumor location (GTR plus STR rates: DMG-H3K27 35.0% vs. DHG-H3G34 75.0% vs. DpHGG-H3wt/IDHwt 75.0% vs. IHG 100.0%, *P* = .001). Adjuvant therapy also differed by diagnosis. Fewer IHG patients received CCRT (46.2%) than did patients with other pHGG subtypes, as radiation was avoided due to the young age of IHG patients. DHG-H3G34 and DpHGG-H3wt/IDHwt were treated with CCRT in 100% and 95.0% of the patients, respectively. DMG-H3K27 was treated with CCRT in 75% of patients and with RT alone in 20% of patients.

### Genomic Characteristics of pHGGs

**Figure 3.**
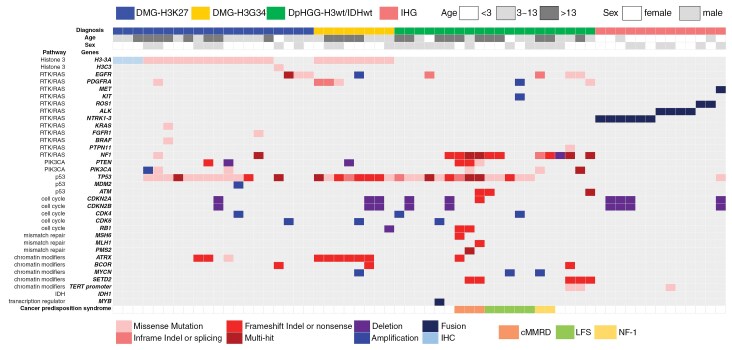
Oncoplot of 61 pHGG patients. The genomic landscape of pHGGs, excluding IHG, revealed that the most common alterations were *TP53* mutations (34/48, 70.8%). All patients with DHG-H3G34 tumors harbored *TP53* mutations (8/8, 100%), while a subset harbored *ATRX* mutations (6/8, 75%). Among patients diagnosed with DpHGG-H3wt/IDHwt, *TP53* mutations were the most common alterations (14/20, 70%), followed by mutations in *NF1* (11/20, 55%) and *SETD2* (5/20, 25%). Three patients previously diagnosed with GC harbored mutations in *SETD2*, *NF1*, the *TERT* promoter and *EGFR* but lacked *TP53* mutations. Cancer predisposition syndromes were noted in DpHGG-H3wt/IDHwt patients and in 3 cMMRD patients, who also had mutations in mismatch repair genes (*MSH6, MLH1*, and *PMS2*) and presented a high mutational burden. Thirteen patients with IHG had 6 *NTRK* fusions, 4 *ALK* fusions, 2 *ROS1* fusions, and 1 *MET* fusion. Abbreviations: pHGG, pediatric-type diffuse high-grade glioma; DMG-H3K27, diffuse midline glioma, H3 K27-altered; DHG-H3G34, diffuse hemispheric glioma, H3 G34-mutant; DpHGG-H3wt/IDHwt, diffuse pediatric-type high-grade glioma, H3-wildtype and IDH-wildtype; IHG, infant-type hemispheric glioma; IHC, immunohistochemistry; cMMRD, constitutional mismatch repair deficiency syndrome; LFS, Li-Fraumeni syndrome; NF-1, neurofibromatosis type 1.

The genomic landscape of pHGGs, excluding IHG (*n* = 13), revealed that the most common alterations were *TP53* mutations (34/48, 70.8%, Figure 3). All patients with DHG-H3G34 harbored *TP53* mutations (8/8, 100%), while only a subset d by *ATRX* mutations (6/8, 75%). Among patients diagnosed with DpHGG-H3wt/IDHwt, *TP53* mutations were the most common alterations (14/20, 70%), followed by mutations in *NF1* (11/20, 55%) and *SETD2* (5/20, 25%). Three patients with DpHGG-H3wt/IDHwt tumors presented mutations in mismatch repair genes (*MSH6*, *MLH1*, and *PMS2*), which are commonly associated with microsatellite instability and a high mutational burden, and thus these mutations may have contributed to tumorigenesis in these patients. Three patients previously diagnosed with GC harbored mutations in *SETD2* (3 patients), *NF1* (2 patients), the *TERT* promoter (2 patients), and *EGFR* (1 patient), but lacked *TP53* mutations.

Thirteen patients with IHGs had 6 *NTRK* fusions, 1 *MET* fusion, 4 *ALK* fusions, and 2 *ROS1* fusions and all but one presented high-grade histopathological features, including a high mitotic rate (range: 5–48/10 HPFs), a high Ki-67 labeling index (range: 10.0–48.0%), and microvascular proliferation or necrosis. One patient who was initially diagnosed with PNET presented a low mitotic rate (1/10 HPFs) and also lacked microvascular proliferation and necrosis.

### Underlying CPS in DpHGG-H3wt/IDHwt

Among the DMG-H3K27, DHG-H3G34, and DpHGG-H3wt/IDHwt patients, 13/20 (76.5%), 8/8 (100%), and 16/20 (80%) met the criteria for germline analysis, respectively (Supplementary Table 2), whereas no IHG cases met the criteria for germline analysis. The association with CPS was significantly greater in DpHGG-H3wt/IDHwt patients, as approximately half of the patients were affected. Germline variants were detected in 0/4 (0.0%) DMG-H3K27 patients, 0/4 (0.0%) DHG-H3G34 patients, and 3/6 (50.0%) DpHGG-H3wt/IDHwt patients, meaning all of the DMG-H3K27 patients and DHG-H3G34 patients were somatic variants. Along with 3 confirmed germline variants in DpHGG-H3wt/IDHwt patients, 7 additional patients also met the clinical diagnostic criteria for LFS, NF-1, or cMMRD. None of the DMG-H3K27 or DHG-H3G34 patients had confirmed germline variants or met the clinical criteria for CPS (0/20 (0.0%) vs. 0/8 (0.0%) vs. 10/20 (50.0%), *P* < .001).

Five patients with LFS, 2 with NF-1, and 3 with cMMRD were identified (Table 2). With the exception of one patient who was diagnosed at 2.3 years of age, all patients with DpHGG-H3wt/IDHwt were teenagers at diagnosis. Eight out of the 10 somatic tumor variants we identified matched the positions of pathogenic or likely pathogenic germline variants listed in the ClinVar database. Two LFS patients developed DpHGG-H3wt/IDHwt adjacent to prior RT fields (Supplementary Figure 4). Among the 5 LFS patients, 3 had family histories that were insufficient to account for their diagnoses, which led to the presumption that these cases might have resulted from de novo mutations. The 2 NF-1 patients met the diagnostic criteria for NF-1, and a germline variant was confirmed in the patient for whom germline analysis was available. Three patients were suspected to have cMMRD based on somatic tumor variants in the *MLH1*, *MSH6*, and *PMS2* genes combined with the high mutational burden observed in their tumors. None of the patients with cMMRD had any significant family history.

**Table 2.** Characteristics of 10 DpHGG-H3wt/IDHwt Patients Suggestive of CPS

No	Sex	Age at surgery	Cancer predisposition syndrome	Location	Family history	Past cancer history or associated lesions	Somatic tumor variant suggestive of germline variant	Germline confirmed	Survival status	Survival period^a^ (months)
1	F	17	LFS	C2–C4 spinal cord	None	Nasopharyngeal rhabdomyosarcoma at 4.2 years RT field adjacent to C-spine	^b^TP53: c.746G>C (p.R249T), VAF: 77.54%	NA	Deceased	19.9
2	M	2.3	LFS	Frontal lobe	Equivocal: 3rd degree relative sarcoma, 4th degree relative brain tumor on father’s side, age unknown	None	^b^TP53: c.586C>T (p.R196^a^), VAF: 73.57%	NA	Deceased	12.3
3	F	13	LFS	Frontal lobe (retro-orbital space)	None	Periorbital rhabdomyosarcoma at 4 years RT field adjacent to frontal lobe	^b^TP53: c.844C>T (p.R282W), VAF: 78.95%	NA	Deceased	66.6
4	F	15	LFS	Temporal lobe	Mother: Leukemia, <45 years	None	TP53: c.1009C>A (p.R337S), VAF: 76.86%	Yes	Alive	17.2
5	M	12	LFS	Frontal lobe	Mother: undifferentiated sarcoma, <45 years	Rib osteochondroma at 6 years	^b^TP53: c.659A>G (p.Y220C), VAF: 95.42%	Yes	Deceased	20.7
6	M	17	NF-1	Frontal lobe	Father: NF-1	Café-au-lait spot Cutaneous neurofibroma	^b^NF1: c.5135delC (p.P1712fs), VAF: 45.57%	NA	Deceased	34.8
7	M	14	NF-1	Temporal lobe-insula	Mother: NF-1	Cafe-au-lait-spot Lisch nodule	^b^NF1: c.1527+1G>T (splicing), VAF: 78.66%	Yes	Alive	40.4
8	F	15	cMMRD	Fronto-temporal lobe	None	None	^b^MLH1: c.808_811delACTT (p.T270fs), VAF: 95.42%	NA	Deceased	11.2
9	M	11	cMMRD	Parietal lobe	None	None	^b^PMS2: c.2444C>T (p.S815L), VAF: 32.39%	NA	Deceased	14.6
10	F	13	cMMRD	Frontal lobe	None	None	MSH6: c.3255_3256delCCinsT (p.P1086fs), VAF: 78.85%	NA	Deceased	14.1

Abbreviations: DpHGG-H3wt/IDHwt, diffuse pediatric-type high-grade glioma, H3-wildtype and IDH-wildtype; LFS, Li-Fraumeni syndrome; NF-1, Neurofibromatosis type 1; cMMRD, constitutional mismatch repair deficiency syndrome; VAF, variant allele frequency; RT, radiotherapy.

^a^Survival period was calculated from the time of surgery.

^b^Pathogenic/Likely Pathogenic germline variant on ClinVar database.

### Outcomes of the pHGG Patients

**Figure 4.**
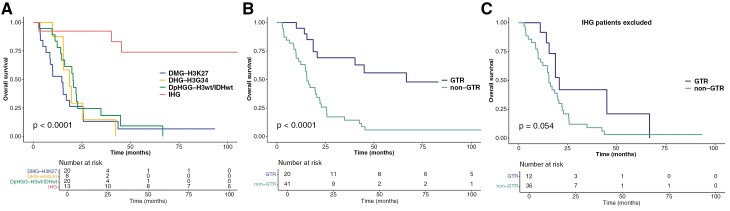
Kaplan–Meier curves for overall survival (OS) of pHGG patients. (A) The 2-year OS rates were 26.3%, 29.2%, 23.2%, and 92.3% for DMG-H3K27, DHG-H3G34, DpHGG-H3wt/IDHwt, and IHG, respectively (*P* < .001). Long-term survival was observed in IHG patients, with 5-year OS rate of 73.8%. (B) GTR was associated with significantly better survival outcomes in pHGG patients overall (*P* < .001). (C) When excluding IHG patients, the 2-year OS rates were 41.9% for those who underwent GTR and 20.9% for those who did not (*P* = .054), suggesting a potential survival benefit when GTR is achieved even in non-IHG pHGG patients. Abbreviations: pHGG, pediatric-type diffuse high-grade glioma; DMG-H3K27, diffuse midline glioma, H3 K27-altered; DHG-H3G34, diffuse hemispheric glioma, H3 G34-mutant; DpHGG-H3wt/IDHwt, diffuse pediatric-type high-grade glioma, H3-wildtype and IDH-wildtype; IHG, infant-type hemispheric glioma; GTR, gross total resection; STR, subtotal resection; PR, partial resection; CPS, cancer predisposition syndrome; LFS, Li-Fraumeni syndrome; NF-1, neurofibromatosis type 1; cMMRD, constitutional mismatch repair deficiency syndrome.

Kaplan–Meier curves revealed 2-year OS rates of 26.3%, 29.2%, 23.2%, and 92.3%, for DMG-H3K27, DHG-H3G34, DpHGG-H3wt/IDHwt, and IHG, respectively (*P* < .001, Figure 4A). No significant differences in OS were observed among the DMG-H3K27, DHG-H3G34, and DpHGG-H3wt/IDHwt patients. Long-term survival was observed in IHG patients, with a 5-year OS rate of 73.8%. GTR was associated with significantly better survival outcomes in pHGG patients overall (*P* < .001) (Figure 4B). Although not statistically significant, a trend towards improved 2-year OS was observed in patients who underwent GTR (41.9%) compared with those who did not (20.9%), suggesting a potential survival benefit even in non-IHG pHGG patients (Figure 4C). No significant difference was seen in OS based on the presence of somatic *NF1*, *TP53*, and *CDKN2A/2B* mutations or the MGMT methylation status (Supplementary Figure 5).

While the clinical characteristics and behavior of IHG are not yet fully understood, this tumor type is well known to have a less aggressive prognosis than other pHGGs. This trend was also observed in our cohort. Among the 3 IHG patients who died, one patient harboring *ALK-*fused IHG died at 45.4 months, and another patient harboring *NTRK1-*fused IHG died at 40.2 months. The third patient who died from IHG presented with a *PTPRZ1::MET* fusion, which may be associated with poorer survival in IHG.^25–27^

## Discussion

BT-targeted NGS using fresh frozen tumor tissues stored for extended periods facilitated diagnostic reclassification in 73 (97.3%) out of 75 patient samples. This high reclassification rate stemmed from significant changes in the WHO classification system. The reclassification of previously diagnosed HGGs and PNETs in children has necessitated a reassessment of clinical data and outcomes. Our findings align with those of previous studies, as a predominant proportion of PNETs were reclassified as ST-EPNs, whereas others were reclassified as pHGGs, including DHG-H3G34, DpHGG-H3wt/IDHwt, and IHG.^28^ Notably, tumors previously classified as pediatric GBMs were reclassified into various pHGG subtypes on the basis of molecular profiling, and some were identified as low-grade gliomas or emerging neuroepithelial tumors, which are characterized by specific genetic alterations, such as fusion genes.

With the increasing use of BT-targeted NGS in pediatric BTs, recent studies have reported the genomic characteristics of pHGGs. DMG-H3K27 and DHG-H3G34 exhibit distinct concurrent mutations.^29^ Mutations in *NF1* and *PIK3CA* are more prevalent in DMG-H3K27, whereas *TP53* and *ATRX* mutations are more characteristic of DHG-H3G34. The diagnosis of DpHGG-H3wt/IDHwt should not be made solely based on the absence of H3 or IDH mutations, as it is not an exclusion diagnosis in pHGGs.^30^ Nevertheless, DpHGG-H3wt/IDHwt comprises heterogeneous molecular features.^31^ Methylation assays of IDH-wildtype GBMs in adult patients frequently reveal that *TP53*, *NF1*, *PTEN*, *SETD2*, and *PDGFRA* mutations are aligned with a diagnosis of DpHGG-H3wt/IDHwt.^32^ The previously defined entity GC has also been rediscovered to comprise heterogeneous subtypes of DpHGG-H3wt/IDHwt, such as the RTK2A/B subtypes.^33^ Considering the presence of *SETD2* and *TERT* promoter mutations without *TP53* mutations, our 3 previously diagnosed GC tumors resemble the pHGG-RTK2A subtype.^34^

Fusion gene detection via transcriptome sequencing facilitated the diagnosis of IHG, ST-EPN, low-grade gliomas, and neuroepithelial tumors, as fusion genes were the only observed driver events in these tumors. Emerging high-grade neuroepithelial tumors with fusion genes involving *CIC, PATZ1,* and *PLAGL1* may be included in future CNS tumor classifications.^35–37^ These findings underscore the importance of fusion gene detection in the diagnosis of pediatric glial and ependymal tumors.^38^

A comprehensive review of medical records, somatic tumor tissue BT-targeted NGS data, and germline sequencing revealed a significant proportion of CPS in patients with pHGGs, particularly in those with DpHGG-H3wt/IDHwt. Histone-mutant tumors, such as DMG-H3K27 and DHG-H3G34, are significantly less strongly associated with CPS despite the high VAF of *TP53* mutations.^39^ While the association between DpHGG-H3wt/IDHwt and CPS (particularly LFS) is gaining recognition, no such associations have been reported for DMG-H3K27 or DHG-H3G34.^40–42^ This feature of DpHGG-H3wt/IDHwt contrasts with previous findings that CPS is relatively rare in patients with pHGGs, whereas BTs such as AT/RT and medulloblastoma should prompt the consideration of CPS.^20,43^ The high proportion of de novo cases and short survival period may have masked CPS and led to its underdetection in individuals with pHGGs. While our study emphasizes the importance of matched germline sequencing, most cases of CPS could still be identified through clinical evaluation. Given the continued limited access to comprehensive genomic sequencing in real-world settings, clinical suspicion, thorough history taking, and physical examination remain important.

For LFS patients with BTs, early imaging surveillance and surgical intervention are recommended.^44^ However, the evidence regarding secondary malignancies and the deleterious effects of RT in LFS patients is conflicting.^45,46^ As observed in our study, radiation-induced malignancies can occur after RT in LFS patients. Recent research suggests that RT should be avoided in LFS patients when alternative treatments are available and recommends the consideration of RT techniques such as particle therapy or dose reduction to minimize the risk of secondary malignancy.^47,48^ Although the pHGG in NF-1 patients were diagnosed as DpHGG-H3wt/IDHwt based on pathological review in our study, methylation profiling should be incorporated to accurately differentiate them from high-grade astrocytoma with piloid features (HGAP).^49,50^ The most common CNS tumor associated with cMMRD, previously categorized as GBM, is now recognized as DpHGG-H3wt/IDHwt.^51^ Significant numbers of cMMRD patients lack a family history consistent with Lynch syndrome, and thus the clinical diagnosis is challenging. Routine-matched germline sequencing will expand our understanding of CPS in patients with pHGGs.^52^

Our analysis revealed distinct demographic and anatomical distribution patterns among pHGG subtypes. IHG demonstrated the most favorable prognosis among the pHGG subtypes. Previously, IHC patients received the same treatment as other pHGG cases. However, reclassifying these diagnoses has prompted us to update our approach to adjuvant therapy. In light of favorable prognosis, it may be appropriate to consider less aggressive and less toxic adjuvant regimens for IHG patients. Additionally, there is increasing evidence supporting the use of targeted therapies in this group.^53–55^ Although not statistically significant, our data suggested a trend towards improved survival in DMG-H3K27, DHG-H3G34, and DpHGG-H3wt/IDHwt patients in whom GTR was achieved. However, it is important to note that the feasibility of GTR is often limited by tumor location, particularly in patients with DMG-H3K27. Despite this challenge, our findings support the pursuit of GTR whenever possible, especially for hemispheric tumors, as it may confer a survival advantage.

This study has several limitations. First, the single-institution design introduces potential selection bias, which may affect the generalizability of our findings. The rarity of other institutes archiving fresh frozen tissue, which poses an obstacle to performing a multicenter study. Second, not all HGGs in the brainstem were biopsed, which may have led to underrepresentation of DMG-H3K27 at our institute. Recently, we have been actively performing biopsies for all gliomas in midline structures, as BT-targeted NGS and diagnosis according to the WHO CNS5 are crucial for accurate patient stratification and treatment planning. Third, the lack of methylation assays for tumor subtyping, particularly for DpHGG-H3wt/IDHwt, limits our ability to further classify these tumors into molecular subgroups. Methylation assays should be available for the diagnosis of these patients, and therefore, we are developing an in-house methylation platform.^56^ Fourth, the targeted nature of our NGS panel limited our ability to accurately assess copy number variations. Finally, this study analyzed both the diagnostic NGS results from past patients and tumor tissue samples that were banked from patients diagnosed before 2018 who did not undergo NGS testing. Because pHGG patients typically have short survival times, it was often not possible to collect normal tissue, such as blood, for germline analysis. These limitations may have led us to underestimate the actual prevalence of CPS in our cohort. In addition to tissue banking, blood sample banking is necessary for genomic analysis in pediatric BT patients.

## Conclusion

The reclassification of previously diagnosed HGGs and PNETs in children based on BT-targeted NGS has facilitated the application of updated classifications according to the WHO CNS5 criteria, providing a more refined understanding of these diagnoses. This process has offered valuable insights into the clinicogenomic characteristics of pHGGs. A notable proportion of patients with DpHGG-H3wt/IDHwt tumors harbored CPS-related mutations. Additionally, the therapeutic approach for IHG is evolving due to their favorable clinical outcomes and the availability of targetable genetic alterations. These observations underscore the importance of accurate molecular diagnosis in patients with pediatric BTs and suggest that treatment strategies should be tailored to specific pHGG subtypes.

## Supplementary Material

vdaf175_suppl_Supplementary_Figure_S1

vdaf175_suppl_Supplementary_Figure_S2

vdaf175_suppl_Supplementary_Figure_S3

vdaf175_suppl_Supplementary_Figure_S4

vdaf175_suppl_Supplementary_Figure_S5

vdaf175_suppl_Supplementary_Materials_1

## Data Availability

The dataset supporting the conclusions of this study is available on request from the corresponding author. The data are not publicly available due to privacy or ethical restrictions.
