# Supplementary figures and images for: Revisiting pediatric HGGs and PNETs according to the WHO CNS5 criteria: A clinical and genomic retrospective analysis
Source: Neurooncol Adv. 2025 Aug 9;7(1):vdaf175. doi: 10.1093/noajnl/vdaf175 (PMC12448711; doi:10.1093/noajnl/vdaf175)

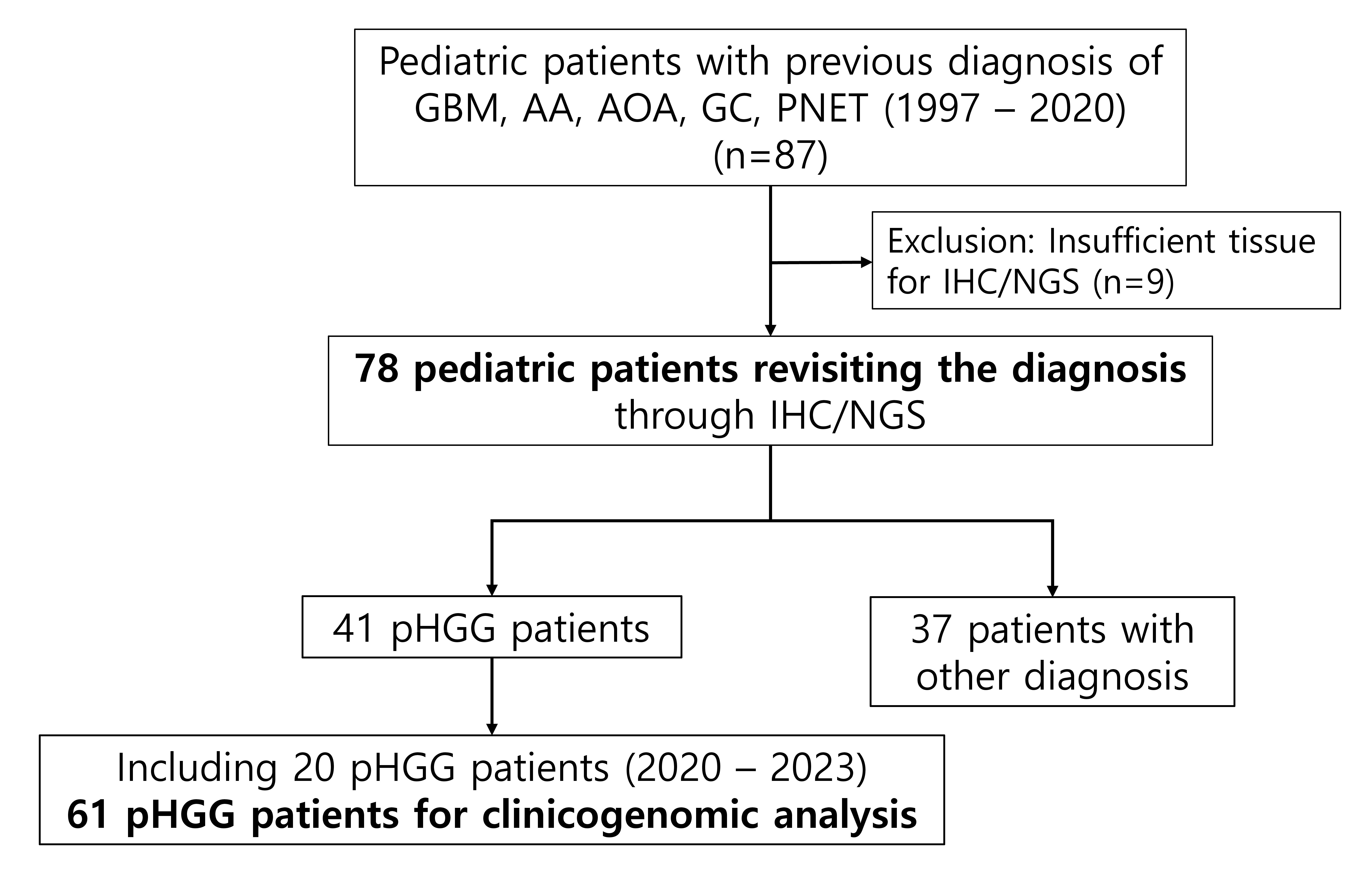

Supplement: vdaf175_suppl_Supplementary_Figure_S1 [file vdaf175_suppl_supplementary_figure_s1.jpeg]

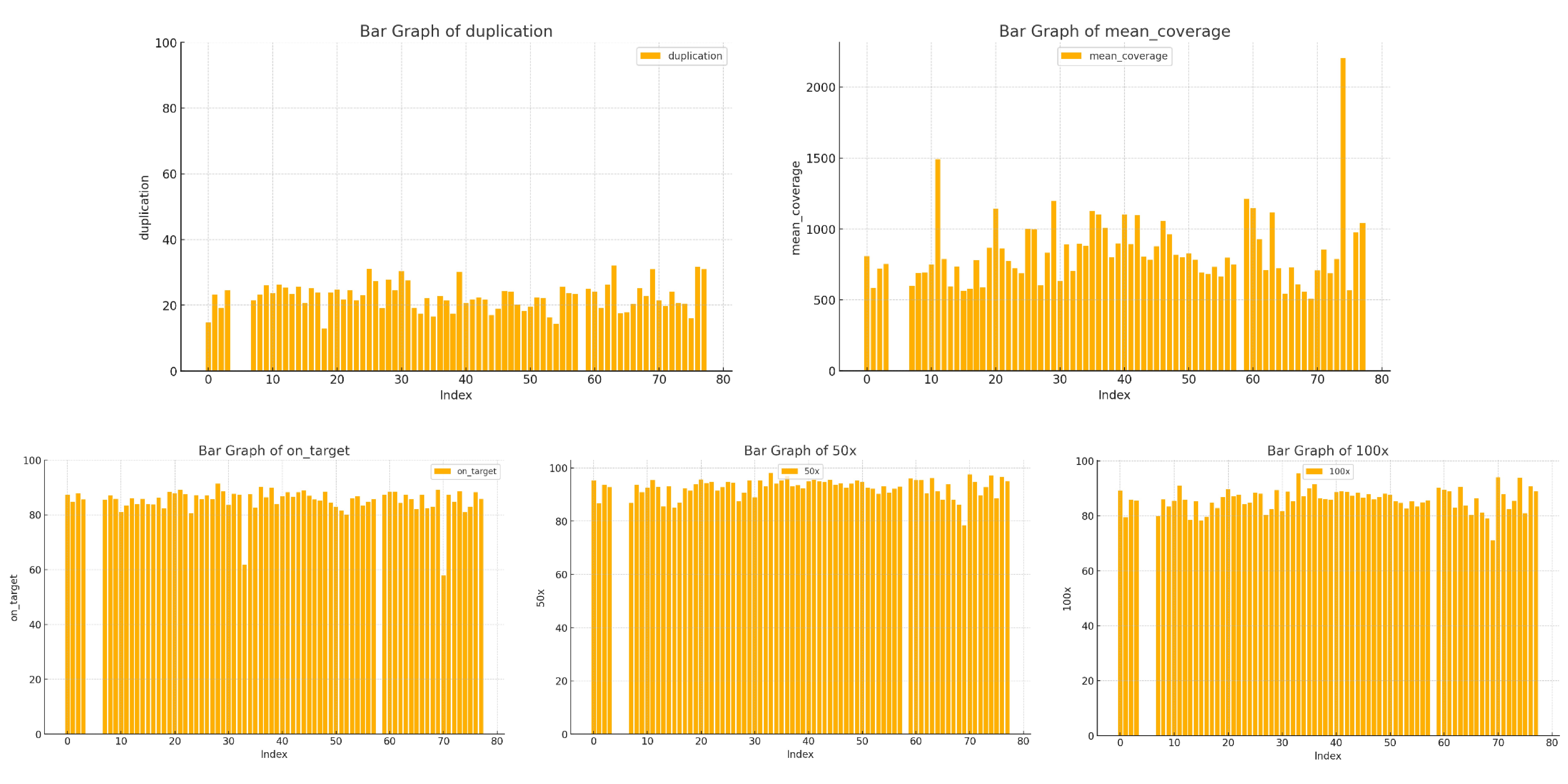

Supplement: vdaf175_suppl_Supplementary_Figure_S2 [file vdaf175_suppl_supplementary_figure_s2.jpeg]

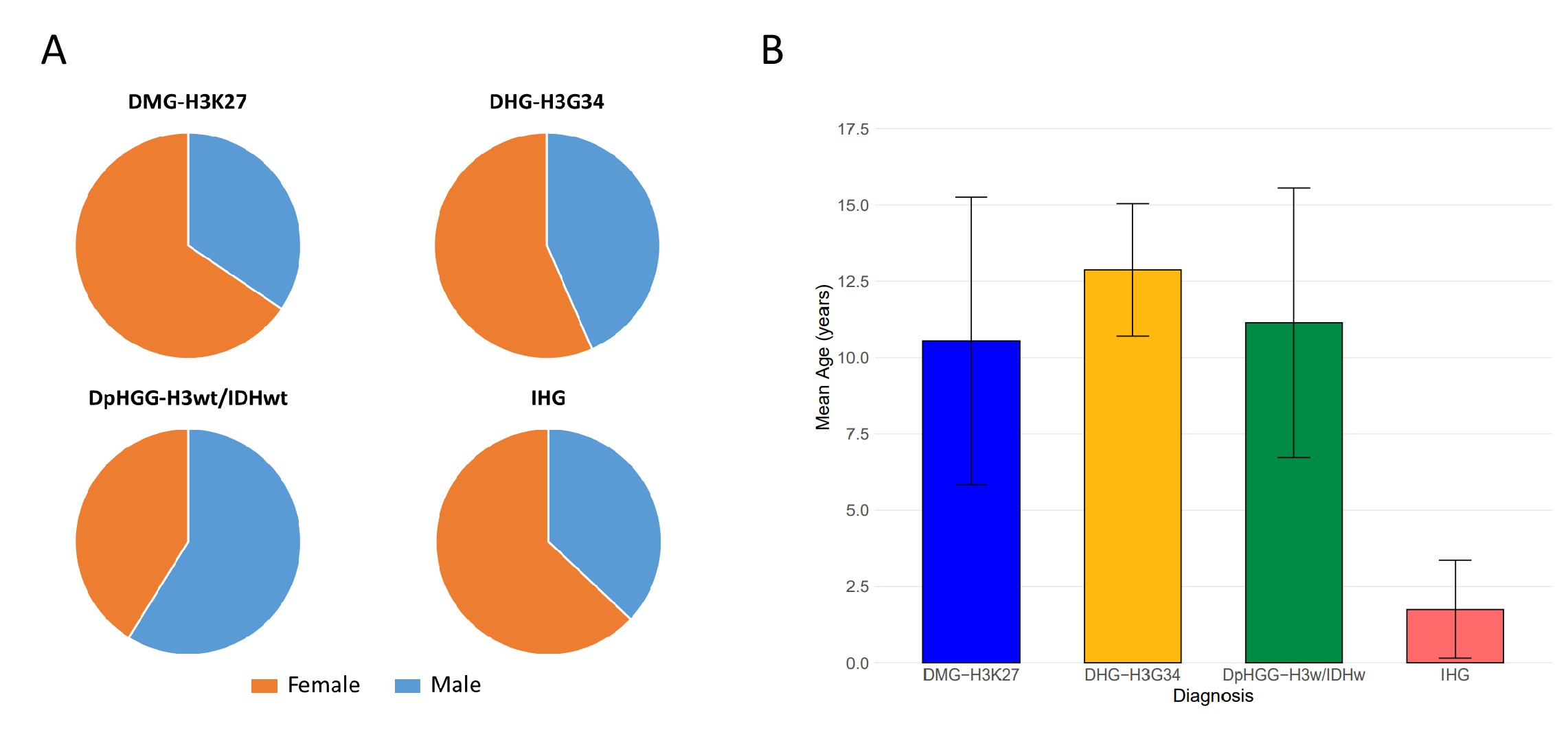

Supplement: vdaf175_suppl_Supplementary_Figure_S3 [file vdaf175_suppl_supplementary_figure_s3.jpeg]

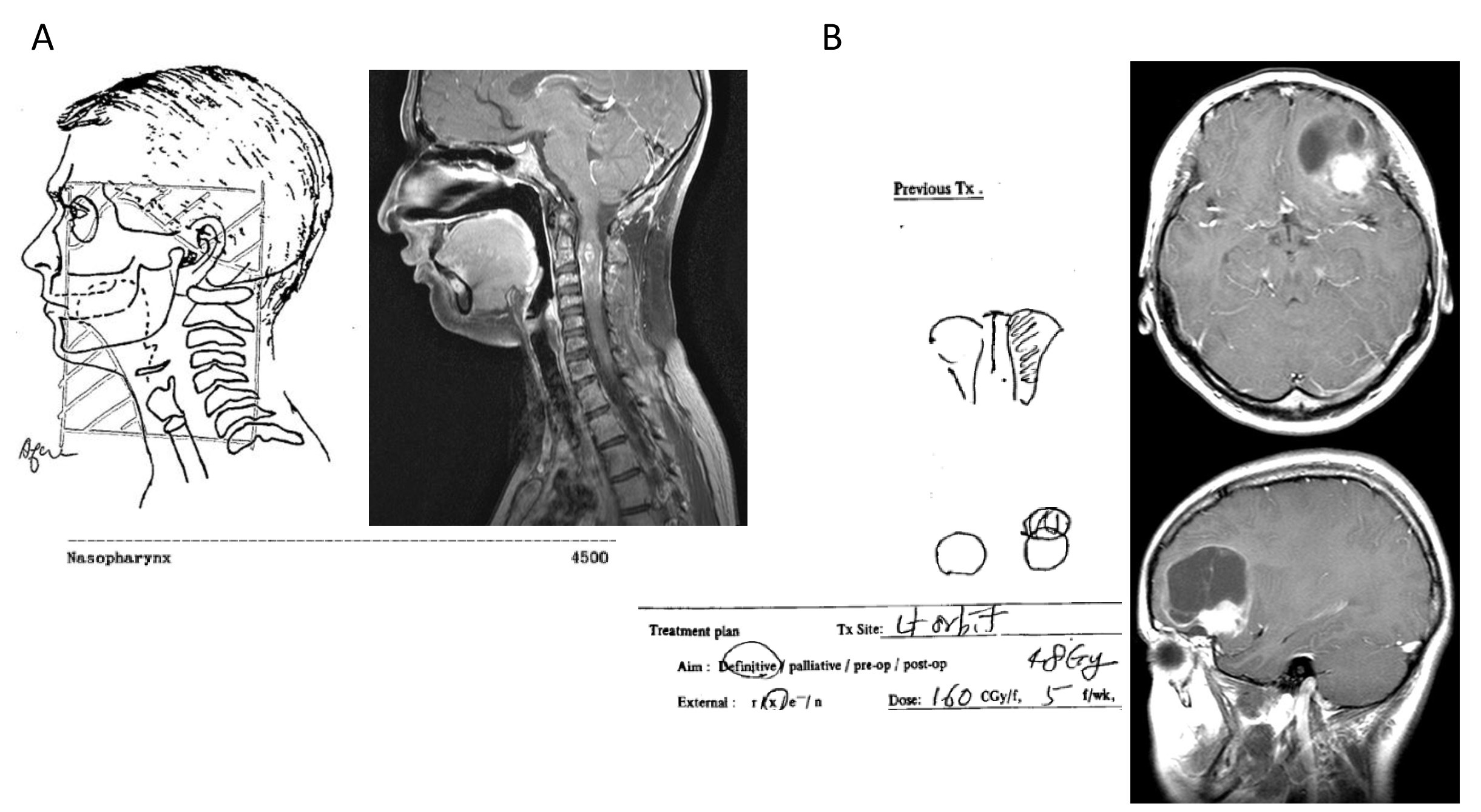

Supplement: vdaf175_suppl_Supplementary_Figure_S4 [file vdaf175_suppl_supplementary_figure_s4.jpeg]

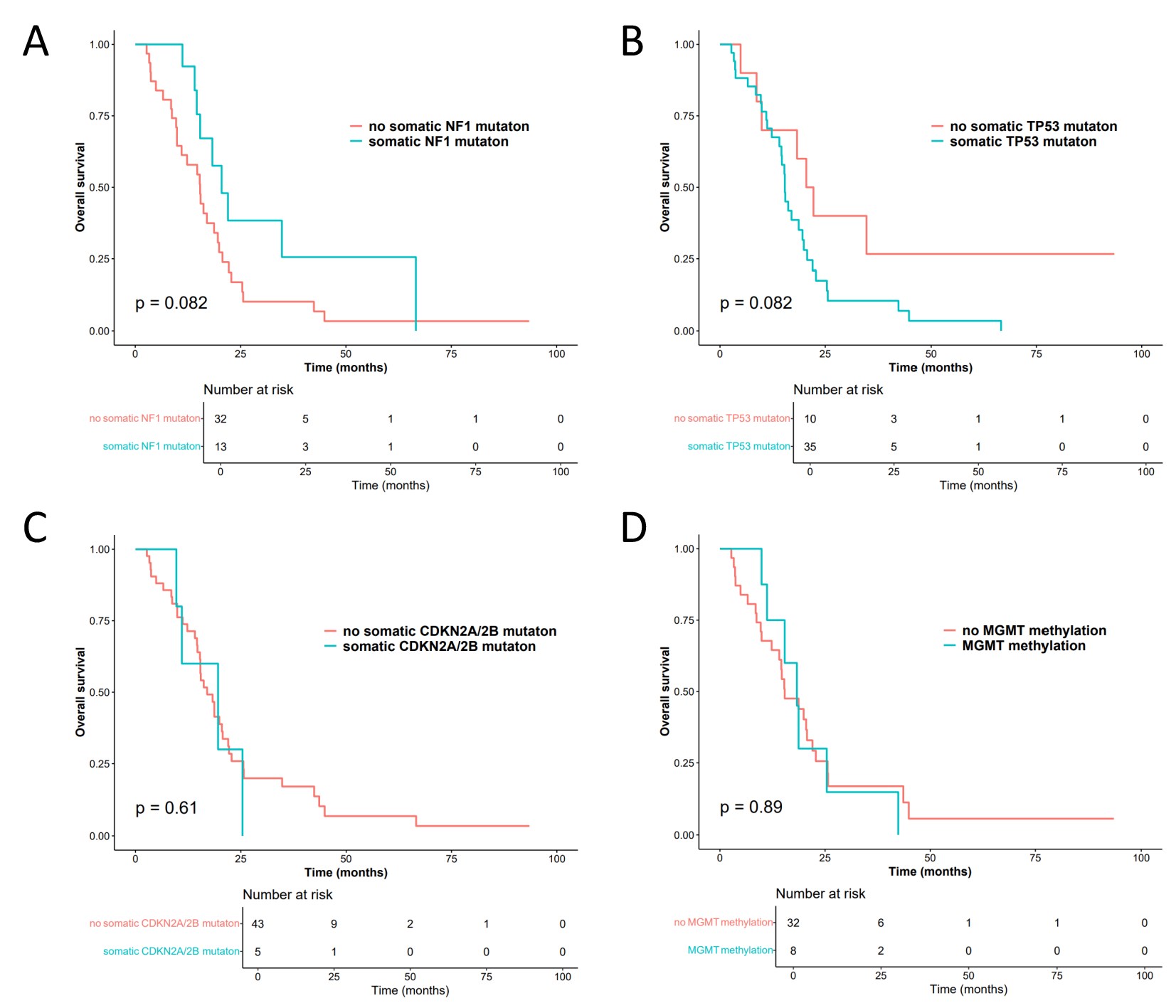

Supplement: vdaf175_suppl_Supplementary_Figure_S5 [file vdaf175_suppl_supplementary_figure_s5.jpeg]
