# Supplementary material for: Revisiting pediatric HGGs and PNETs according to the WHO CNS5 criteria: A clinical and genomic retrospective analysis
Source: Neurooncol Adv. 2025 Aug 9;7(1):vdaf175. doi: 10.1093/noajnl/vdaf175 (PMC12448711; doi:10.1093/noajnl/vdaf175)
Supplement: vdaf175_suppl_Supplementary_Materials_1 [file vdaf175_suppl_supplementary_materials_1.docx]

**Supplementary Table 1. The list of the primary antibodies used in this study.**

| Antibody | Dilution | Antigen retrieval | Clone | Source |
| --- | --- | --- | --- | --- |
| ATRX | 1:300 | Ventana CC1 at 100^o^C | Polyclonal | Atlas Antibodies, AB, Bromma, Sweden |
| GFAP | 1:200 | Ventana CC1 at 100^o^C | 6F2 (monoclonal) | DAKO, Glostrup, Denmark |
| H3K27M | 1:1,000 | Ventana CC1 at 100^o^C | Polyclonal | Millipore, Temecula, CA |
| H3K27me3 | 1:100 | Ventana CC1 at 100^o^C | C36B11 (monoclonal) | Cell Signaling, Boston, MA |
| Synaptophysin | 1:200 | Ventana CC1 at 100^o^C | 27G12 (monoclonal) | Novocastra, Newcastle, UK |
| IDH1 | 1:100 | Ventana CC1 at 100^o^C | H09 (monoclonal) | Dianova, Hamburg, Germany |
| INI-1 | 1:100 | Ventana CC1 at 100^o^C | MRQ27 (monoclonal) | Cell Marque, Rocklin, CA |
| Ki67 | 1:1,000 | Ventana CC1 at 100^o^C | M7240 (monoclonal) | DAKO, Glostrup, Denmark |
| L1CAM | 1:10,000 | Ventana CC1 at 100^o^C | UJ127 (monoclonal) | Millipore, Temecula, CA |
| NFkB | 1:1,000 | Ventana CC1 at 100^o^C | D14E12 (monoclonal) | Cell Signaling, Boston, MA |
| P53 | 1:100 | Ventana CC1 at 100^o^C | DO7 (monoclonal) | DAKO, Glostrup, Denmark |
| pHH3 | 1:100 | Ventana CC1 at 100^o^C | 369A-15 (polyclonal) | Cell Marque, Rocklin, CA |

GFAP, glial fibrillary acidic protein; K27M, antibody for Histone 3 Lys27Met; pHH3, phosphorylated Histone H3.

**Supplementary Table 2. Germline analysis and clinically suspected CPS in pHGG subtypes**

|  | DMG-H3K27  (n=20) | DHG-H3G34  (n=8) | DpHGG-H3wt/IDHwt  (n=20) | p value |
| --- | --- | --- | --- | --- |
| Somatic NGS results suggesting germline analysis | 13/20 (76.5%) | 8/8 (100.0%) | 16/20 (80.0%) | 0.336 |
| Germline analysis performed | 4/13 (30.8%) | 4/8 (50.0%) | 6/16 (37.5%) | 0.677 |
| Germline variant confirmed | 0/4 (0.0%) | 0/4 (0.0%) | 3/6 (50.0%) | 0.078 |
| Germline variant confirmed and/or clinically suspected CPS | 0/20 (0.0%) | 0/8 (0.0%) | 10/20 (50.0%) | < 0.001 |

DMG-H3K27: diffuse midline glioma, H3 K27-altered; DHG-H3G34: Diffuse hemispheric glioma, H3 G34-mutant; DpHGG-H3wt/IDHwt: diffuse pediatric-type high-grade glioma, H3-wildtype and IDH-wildtype; NGS: next generation sequencing; CPS: cancer predisposition syndrome.

Supplementary Figure 1. Patient flow chart for this study.

A total of 78 pediatric patients diagnosed with high-grade gliomas and PNETs before WHO CNS5 underwent reclassification of diagnosis based on IHC and NGS. To enhance the analysis of the clinical characteristics of pHGGs, 20 additional patients diagnosed with pHGGs after WHO CNS5 were included. Sixty-one pHGG patients were included in the clinicogenomic analysis.

GBM: glioblastoma, AA: anaplastic astrocytoma, AOA: anaplastic oligoastrocytoma, GC: gliomatosis cerebri, PNET: primitive neuroectodermal tumor, IHC: immunohistochemistry, NGS: next-generation sequencing, pHGG: pediatric-type diffuse high-grade glioma, WHO CNS5: The 2021 WHO Classification of Tumors of the Central Nervous System, 5th edition.

Supplementary Figure 2. Quality metrics (duplication rate, coverage depth, and on-target rate) of NGS.
NGS: next-generation sequencing

Supplementary Figure 3. (A) Gender and (B) age distributions of pHGG patients.

The male-to-female ratios were as follows: 1:1.9 in DMG-H3K27 patients, 1:1.3 in DHG-H3G34 patients, 1:0.7 in DpHGG-H3wt/IDHwt patients, and 1:1.7 in IHG patients. The mean age at diagnosis was 10.6 ± 4.7 years in DMG-H3K27 patients, 12.9 ± 2.2 years in DHG-H3G34 patients, 11.2 ± 4.4 years in DpHGG-H3wt/IDHwt patients, and 1.8 ± 1.6 years in IHG patients.

pHGG: pediatric-type diffuse high-grade glioma, DMG-H3K27: diffuse midline glioma, H3 K27-altered, DHG-H3G34: diffuse hemispheric glioma, H3 G34-mutant, DpHGG-H3wt/IDHwt: diffuse pediatric-type high-grade glioma, H3-wildtype and IDH-wildtype, IHG: infant-type hemispheric glioma.

Supplementary Figure 4. Radiation fields and occurrence of DpHGG-H3wt/IDHwt in two LFS patients. (A) The first patient received RT for nasopharyngeal rhabdomyosarcoma at 4.2 years (left) and developed cervical spine DpHGG-H3wt/IDHwt at 17 years (right). (B) The second patient received RT for periorbital rhabdomyosarcoma at 4 years (left) and developed frontal lobe DpHGG-H3wt/IDHwt at 13 years (right).
DpHGG-H3wt/IDHwt: diffuse pediatric-type high-grade glioma, H3-wildtype and IDH-wildtype, LFS: Li-Fraumeni syndrome; RT: radiotherapy.

Supplementary Figure 5. Kaplan–Meier curves for pHGGs excluding IHG, for (A) *NF1*, (B) *TP53*, (C) *CDKN2A/2B* somatic mutations and (D) the MGMT methylation status.
pHGG: pediatric-type diffuse high-grade glioma; IHG: infant-type hemispheric glioma.
